# The Role of Numeracy, Literacy, and Education on Cognition Among Indigenous and Non-Indigenous Mexican Older Adults

**DOI:** 10.1093/geroni/igaf122.1515

**Published:** 2025-12-31

**Authors:** Sirena Gutierrez, Iris Strangmann, Zachary Kunicki, Alexa Gonzalez, Gelan Ying, Jet Vonk, Emily Briceño, Miguel Rentería

**Affiliations:** University of California, San Francisco, San Francisco, California, United States; Columbia University Medical Center, New York, New York, United States; Warren Alpert Medical of Brown University, Providence, Rhode Island, United States; University of Houston, Houston, Texas, United States; University of Florida, Gainesville, Florida, United States; UCSF Memory and Aging Center, San Francisco, California, United States; University of Michigan Medical School, Ann Arbor, Michigan, United States; Taub Institute for Research on Alzheimer’s Disease and the Aging Brain, Columbia University College of Physicians and Surgeons, New York, New York, United States

## Abstract

Bilingualism is generally associated with better cognitive performance; however, Indigenous-Spanish bilingual older adults tend to perform worse than their non-Indigenous monolingual counterparts. Older Indigenous populations in Mexico, particularly in rural areas, face inequities in education, healthcare access, and pervasive discrimination. It is unclear if factors beyond education, like literacy and numeracy, influence brain aging in Indigenous adults. This analysis included 1,958 adults ages 55 + (mean 68.1 [8.9±SD] years) from the Mexican Health and Aging Study Ancillary Study on Cognitive Aging (2016). Indigeneity was assessed via self-reported indigenous language spoken. Linear regression models estimated the relationship between speaking an indigenous language and cognitive function factor scores, adjusting for early-life sociodemographic covariates. Mediation analysis decomposed the effects of Indigeneity into direct and indirect effects through numeracy, literacy, and education. Among participants, 11% spoke an indigenous language. These individuals had lower educational attainment (3.2 vs. 5.7 years) and were more likely to reside in rural areas (71.4% vs. 39.6%). Speaking an indigenous language was associated with lower memory (β[95% CI]= -0.31 [-0.40, -0.21]), language (-0.65 [-0.75, -0.55]), executive function (-0.60 [-0.73, -0.47]), and orientation (-0.31 [-0.44, -0.18]) scores. Numeracy mediated 2%-9%, literacy 11%-26%, and education 31%-61% of the total effect of speaking an indigenous language on domain-specific cognition. Numeracy and literacy may be important mechanisms underlying cognitive disparities, alongside education, and efforts to improve them could help reduce these gaps. Future research should explore other factors, such as health conditions and racism, contributing to poorer cognitive outcomes among Indigenous populations in Mexico.

